# Undetected pseudoprogressions in the CeTeG/NOA-09 trial: hints from postprogression survival and MRI analyses

**DOI:** 10.1007/s11060-023-04444-x

**Published:** 2023-09-20

**Authors:** Thomas Zeyen, Daniel Paech, Johannes Weller, Niklas Schäfer, Theophilos Tzaridis, Cathrina Duffy, Louisa Nitsch, Matthias Schneider, Anna-Laura Potthoff, Joachim Peter Steinbach, Peter Hau, Uwe Schlegel, Clemens Seidel, Dietmar Krex, Oliver Grauer, Roland Goldbrunner, Pia Susan Zeiner, Ghazaleh Tabatabai, Norbert Galldiks, Walter Stummer, Elke Hattingen, Martin Glas, Alexander Radbruch, Ulrich Herrlinger, Christina Schaub

**Affiliations:** 1 Division of Clinical Neurooncology, Department of Neurology, University Hospital Bonn, Bonn, Germany; 2 Department of Neuroradiology, University Hospital Bonn, Bonn, Germany; 3 Department of Neurosurgery, University Hospital Bonn, Bonn, Germany; 4 Dr. Senckenberg Institute of Neurooncology, University of Frankfurt, Frankfurt, Germany; 5 Department of Neurology and Wilhelm Sander NeuroOncology Unit, University Hospital Regensburg, Regensburg, Germany; 6 Department of Neurology, Klinik Hirslanden, Zürich, Switzerland; 7 Department of Radiation Oncology, University of Leipzig, Leipzig, Germany; 8 Department of Neurosurgery, Technische Universität Dresden, Faculty of Medicine and University Hospital Carl Gustav Carus, Fetscherstrasse 74, 01307 Dresden, Germany; 9 Department of Neurology, University of Münster, Münster, Germany; 10 Center of Neurosurgery Department of General, Neurosurgery University of Cologne, Cologne, Germany; 11 Department of Neurology and Interdisciplinary Neuro-Oncology, Institute for Clinical Brain Research, University Hospital Tübingen, Eberhard Karls University Tübingen, HertieTübingen, Germany; 12 Center for Neuro-Oncology, Comprehensive Cancer Center Tübingen-Stuttgart, University Hospital Tübingen, Eberhard Karls University Tübingen, Tübingen, Germany; 13 Department of Neurology, Faculty of Medicine and University Hospital Cologne, University of Cologne, Cologne, Germany and Research Center Juelich, Inst. of Neuroscience and Medicine (INM-3), Juelich, Germany; 14 Department of Neurosurgery, University of Münster, Münster, Germany; 15 Department of Neuroradiology, University Hospital Frankfurt, 60590 Frankfurt Am Main, Germany; 16 Division of Clinical Neurooncology, Department of Neurology and Center for Translational Neuro- and Behavioral Sciences (C-TNBS), University Medicine Essen, University Duisburg-Essen, Essen, Germany; 17 German Cancer Consortium (DKTK), Partner Site University Medicine Essen, Hufelandstr. 55, 45147 Essen, Germany

**Keywords:** Glioblastoma, MGMT promotor methylation, Progression, Pseudoprogression, MRI

## Abstract

**Purpose:**

In the randomized CeTeG/NOA-09 trial, lomustine/temozolomide (CCNU/TMZ) was superior to TMZ therapy regarding overall survival (OS) in MGMT promotor-methylated glioblastoma. Progression-free survival (PFS) and pseudoprogression rates (about 10%) were similar in both arms. Further evaluating this discrepancy, we analyzed patterns of postprogression survival (PPS) and MRI features at first progression according to modified RANO criteria (mRANO).

**Methods:**

We classified the patients of the CeTeG/NOA-09 trial according to long vs. short PPS employing a cut-off of 18 months and compared baseline characteristics and survival times. In patients with available MRIs and confirmed progression, the increase in T_1_-enhancing, FLAIR hyperintense lesion volume and the change in ADC mean value of contrast-enhancing tumor upon progression were determined.

**Results:**

Patients with long PPS in the CCNU/TMZ arm had a particularly short PFS (5.6 months). PFS in this subgroup was shorter than in the long PPS subgroup of the TMZ arm (11.1 months, p = 0.01). At mRANO-defined progression, patients of the CCNU/TMZ long PPS subgroup had a significantly higher increase of mean ADC values (p = 0.015) and a tendency to a stronger volumetric increase in T_1_-enhancement (p = 0.22) as compared to long PPS patients of the TMZ arm.

**Conclusion:**

The combination of survival and MRI analyses identified a subgroup of CCNU/TMZ-treated patients with features that sets them apart from other patients in the trial: short first PFS despite long PPS and significant increase in mean ADC values upon mRANO-defined progression. The observed pattern is compatible with the features commonly observed in pseudoprogression suggesting mRANO-undetected pseudoprogressions in the CCNU/TMZ arm of CeTeG/NOA-09.

**Supplementary Information:**

The online version contains supplementary material available at 10.1007/s11060-023-04444-x.

## Introduction

Pseudoprogression is a well-known and frequently occurring phenomenon in glioblastoma patients and has considerable clinical relevance [1–3]. It is defined as MRI changes that mimic tumor progression and eventually resolve or remain stable without change of therapy or can be histologically confirmed as reactive changes without evidence of proliferating tumor. Despite its clinical relevance, it is an insufficiently understood phenomenon and is under further investigation [4, 5].

The exact frequency of pseudoprogression is not clear, current literature describes incidences of 10–30% [6, 7]; transiently increased contrast uptake after radiation even develops in up to 50% [2]. Some authors also describe pseudoprogression as a potential surrogate marker of treatment efficacy, especially in patients with MGMT promotor methylation, although this is still controversial [8–10]. MRI perfusion imaging and amino acid positron-emission-tomography (PET) imaging are used in routine clinical practice to detect pseudoprogression [11]. Furthermore, novel imaging approaches, such as amide proton transfer (APT)-weighted MRI showed promising results in the differentiation of therapy-related changes and tumor progression [12–15]. The apparent diffusion coefficient (ADC) can be derived from diffusion-weighted imaging (DWI) and has been shown to aid distinguishing between true progression and pseudoprogression as higher ADC values in T_1_-enhancement tissue might indicate the latter one [16–19].

In the randomized CeTeG/NOA-09 trial [20], combined lomustine (CCNU)/TMZ was superior to TMZ therapy regarding OS in newly diagnosed patients with MGMT-methylated glioblastoma. Despite the OS benefit, PFS and pseudoprogression rates did not differ significantly between treatments. For progression assessment, (modified) RANO criteria were used in this study similar to most clinical trials investigating glioma therapy [21]. Beyond the limit of 12 weeks after the end of radiotherapy, mRANO criteria allow to assume pseudoprogression only if the suspected contrast-enhancing lesion remains stable or decreases in a follow-up MRI within 8 weeks. However, late and prolonged pseudoprogression [22–24] that does not show stabilization on the first control MRI may thus go undetected.

In this study, we investigated the hypothesis that undetected pseudoprogressions might be accountable for at least some of the discrepancy for the lack of a PFS-prolonging effect in the CeTeG/NOA-09 trial despite OS prolongation. Hypothesizing that the probability of an undetected pseudoprogression increases in patients who have a particularly short first PFS and a very long postprogression survival (PPS), we analysed patterns of PPS and MRI features including tumor volumetry and ADC analysis at the mRANO-defined progression time point.

## Methods

### Study design

**Fig. 1:**
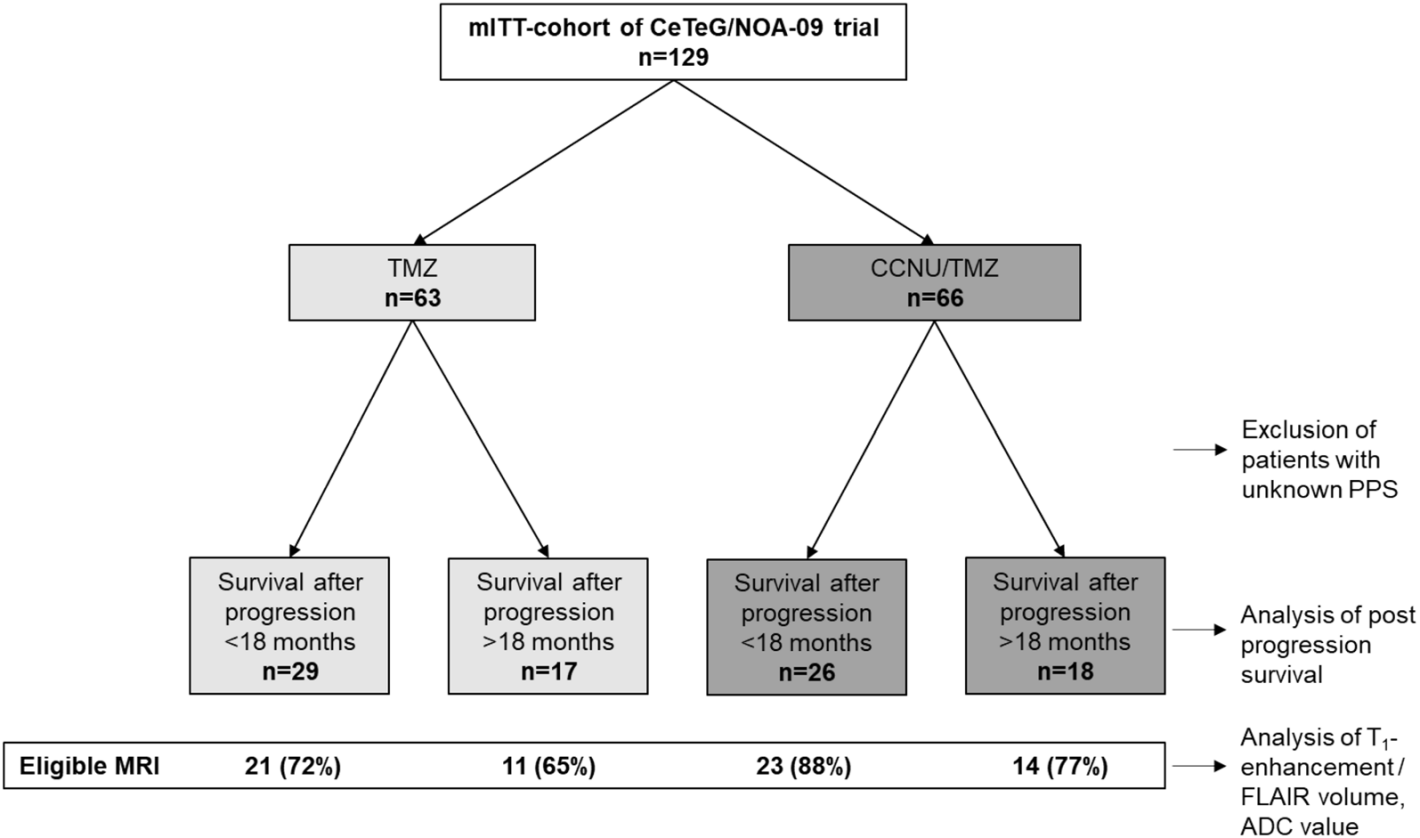
Flowchart of patient identification. Flowchart shows patient selection and identification evaluating patients from the modified intention-to-treat population of the CeTeG/NOA-09 trial. First, subdivision was made by TMZ vs CCNU/TMZ therapy, second subdivision was made by post progression survival with a cut-off of 18 months (defined as survival after progression diagnosis according to mRANO criteria). In this step, patients with unknown PPS (censored PFS and/or censored OS, respectively) were excluded and did not enter any of the following analyses

The prospective, randomized, controlled CeTeG/NOA-09 trial (EudraCT-2009–011252-22, Herrlinger et al. [20]) included 129 patients in the intention-to-treat cohort. Patients were randomized (1:1) to either CCNU/TMZ combination therapy or TMZ standard therapy (Fig. 1). Contrast-enhanced cranial MRI were performed every 12 weeks. We included all patients with disease progression according to mRANO. For patients with a censored PPS below 18 months, subgroup allocation to long vs. short PPS was impossible and these patients were excluded from the analysis. Patients of this trial entered the current MRI-based analyses, if their MRIs were evaluable for T_1_-enhancement and FLAIR volumetry at the time point of progression and, for comparison, at the last MRI prior to progression. Analyses were performed by an independent neuroradiologist. For determination of progression, the modified RANO criteria [20] were used: up to 12 weeks after completion of radiotherapy, disease progression was considered only for new enhancing lesions outside the radiation field (beyond the 80% isodose) or unequivocal histological demonstration of proliferating tumor. According to previous experience with late pseudoprogression, disease progression 12 to 24 weeks after completion of radiotherapy could only be diagnosed if another MRI showing further progression confirmed it 4–6 weeks afterwards. Figure 1 shows the patient selection process for this analysis in a flowchart. In both arms, patients were further subdivided into those with short PPS (defined as ≤ 18 months) and long PPS (> 18 months). A PPS/PFS ratio was calculated for each patient.

## MRI evaluation

### Tumor volume assessment

Manual evaluation of contrast-enhancing and FLAIR hyperintensity volume was performed by using the Medical Imaging Interaction Toolkit software (MITK, Workbench and Toolkit 2016.11, provided by the German Cancer Research Center (DKFZ)). MRI data were performed in 1.5 or 3 T scanners. In this multicenter study cohort, MRIs were conducted in the scanners of the respective centers. The tumor volume was outlined on Gadolinium-enhanced T_1_ MRI data. Measurement of T_1_-enhancement volume (solid tumor) and FLAIR volume (solid tumor and edema) were performed separately for each patient and time point for volumetric assessment. For the T_1_-enhancing volume, the inner necrotic zone has been subtracted (solid tumor volume = T_1_-enhancement volume – volume of necrosis zone). In supplementary Fig. 1A, a3D reconstruction example of tumor segmentation is shown. The tumor volume, that is defined as “region of interest” (ROI) is shown in red.

### ADC analysis

All imaging data were co-registered performed using the “multimodal.rigid.default” registration algorithm in the Medical Imaging Interaction Toolkit [25]. Subsequently, the combined ROI of FLAIR and T_1_-enhancement volume was used to determine the mean ADC value from both baseline and progression time point in the tumor region (Supplementary Fig. 1B). For comparison between treatment groups, the absolute change of Mean ADC-value from baseline to progression time point was compared between patients with short PPS and long PPS.

### Statistical analysis

Statistical analyses have been performed using SPSS (IBM software, Version 27). Analyses of OS, PFS and PPS have been performed according to Kaplan–Meier with a two-sided log-rank test for significance. In contrast to the primary planned confirmatory analysis of CeTeG/NOA-09 trial, which required a log-rank test with stratification by center and recursive partitioning analysis (RPA) class, the analyses in the current report were performed without stratification due to the relatively low number of patients in the subgroups making stratified analyses unapplicable. Median OS, PFS and PPS are reported with a 95% confidence interval (CI).

For comparing PPS/PFS ratios between subgroups we performed a rank sum test (Mann–Whitney-test).Mann–Whitney-test was also performed analyzing tumor/edema volumetry and ADC mean values. Kruskal-Walis-test was performed for comparing median ages and median Karnofsky score between all subgroups. For comparing achieved gross total resections (GTR %) and frequency of second line therapies we used a chi^2^-test.

For all statistical analyses, p-values of < 0.05 were regarded statistically significant. In the figures, significant results are marked as * =  < 0.05, ** =  < 0.005. In selected cases, the p-values are shown within the figure; otherwise, they can be found in the figure legend or the manuscript text.

## Results

For analysis of post progression survival the modified intention to treat population was used after exclusion of patients that had unknown PPS (n = 90/129 patients). In the TMZ arm, 29 evaluable patients had short PPS and 17 had long PPS (≥ 18 months), in the CCNU/TMZ arm 26 evaluable patients had short PPS and 18 had long PPS (Fig. 1). The median age, rate of gross total resections and the median KPS were similar in the four subgroups (Table 1). Also, there were no significant differences in the use of further line therapies between the long PPS CCNU/TMZ and the long PPS TMZ group (Supplementary Fig. 2).

**Table 1:** Median Karnofsky score, age and percentage of gross total resection (GTR) in subgroups

Therapy	TMZ		CCNU/TMZ		*p*-value
PPS	< 18 months	> 18 months	< 18 months	> 18 months	
Number of patients	29	17	26	18	*–*
Median KPS [%] (range)	90 (70–100)	100 (70–100)	100 (70–100)	90 (80–100)	0.79
Median age [years] (range)	57 46–71)	58 (31–70)	54 (41–69)	56 (28–69)	0.08
GTR [%]	72.4	47.1	61.5	61.1	0.38

For each group median Karonfsky score (KPS), median age and extend of resection as percentage of gross total resections (GTR) is given. For analysis of KPS and age rangsum-test (Kruskal-Walis) was performed. GTR (%) was compared by X^2^ test

**Fig. 2:**
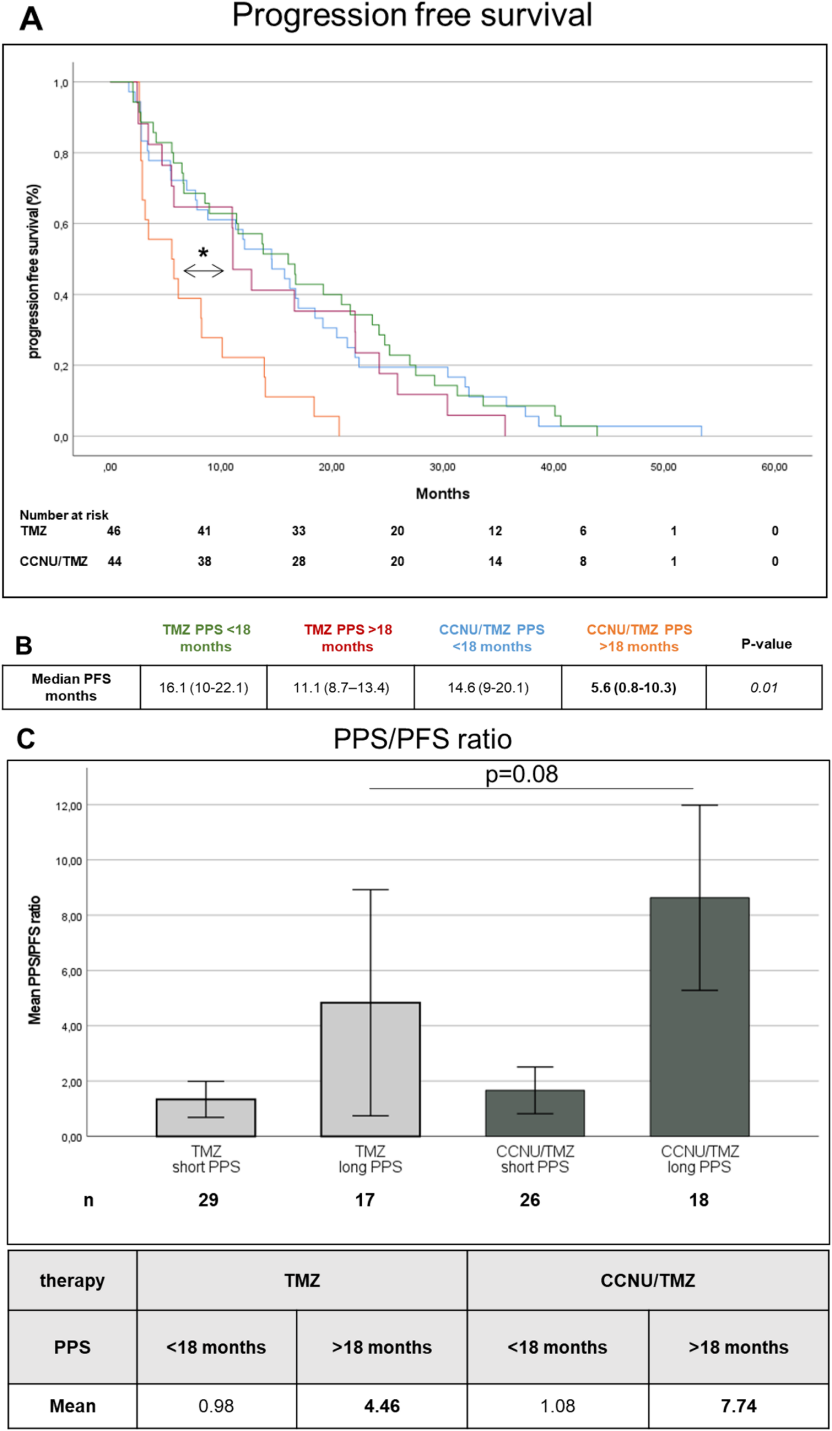
Decreased progression-free survival and higher PPS/PFS ratio in long PPS CCNU/TMZ patients. Kaplan–Meier plots of patients followed TMZ or CCNU/TMZ therapy. Progression free survival (**A**) is given in patients from the modified intention to treat cohort of CeTeG/NOA-09 trial that had known PPS. Subdivision was made in short post progression survival (< 18 months) and long post progression survival (> 18 months) groups. Median PFS is remarkably low in CCNU/TMZ long PPS group compared to TMZ long PPS group (**B**), p = 0.01 (log-rank test). (**C**) Graph shows Mean PPF/PFS ratio of each subgroup

PFS in the long PPS group of the CCNU/TMZ arm was remarkably shorter as compared to the long PPS group of the TMZ arm (median 5.6 months vs 11.1 months, p = 0.01, Fig. 2A). Going in line with this, the mean PPS/PFS ratio tended to be higher in the long PPS group of the CCNU/TMZ as opposed to the long PPS group of the TMZ arm (7.7 vs. 4.5, p = 0.08, Fig. 2C). The PPS/PFS ratio was similarly low in the short PPS subgroups of TMZ and CCNU/TMZ arms with short PPS (1 vs. 1.1, p = 0.632).

The combination of particularly low PFS with very long OS is prominently seen in the long PPS subgroup of the CCNU/TMZ arm. This raises the question whether at the time point of fulfilling the mRANO progression criteria, the underlying biology (e.g. contribution of pseudoprogression) in this subgroup may be different from the long PPS subgroup of the TMZ arm. We further investigated if these differences are also mirrored in MRI at mRANO-defined progression.

**Fig. 3:**
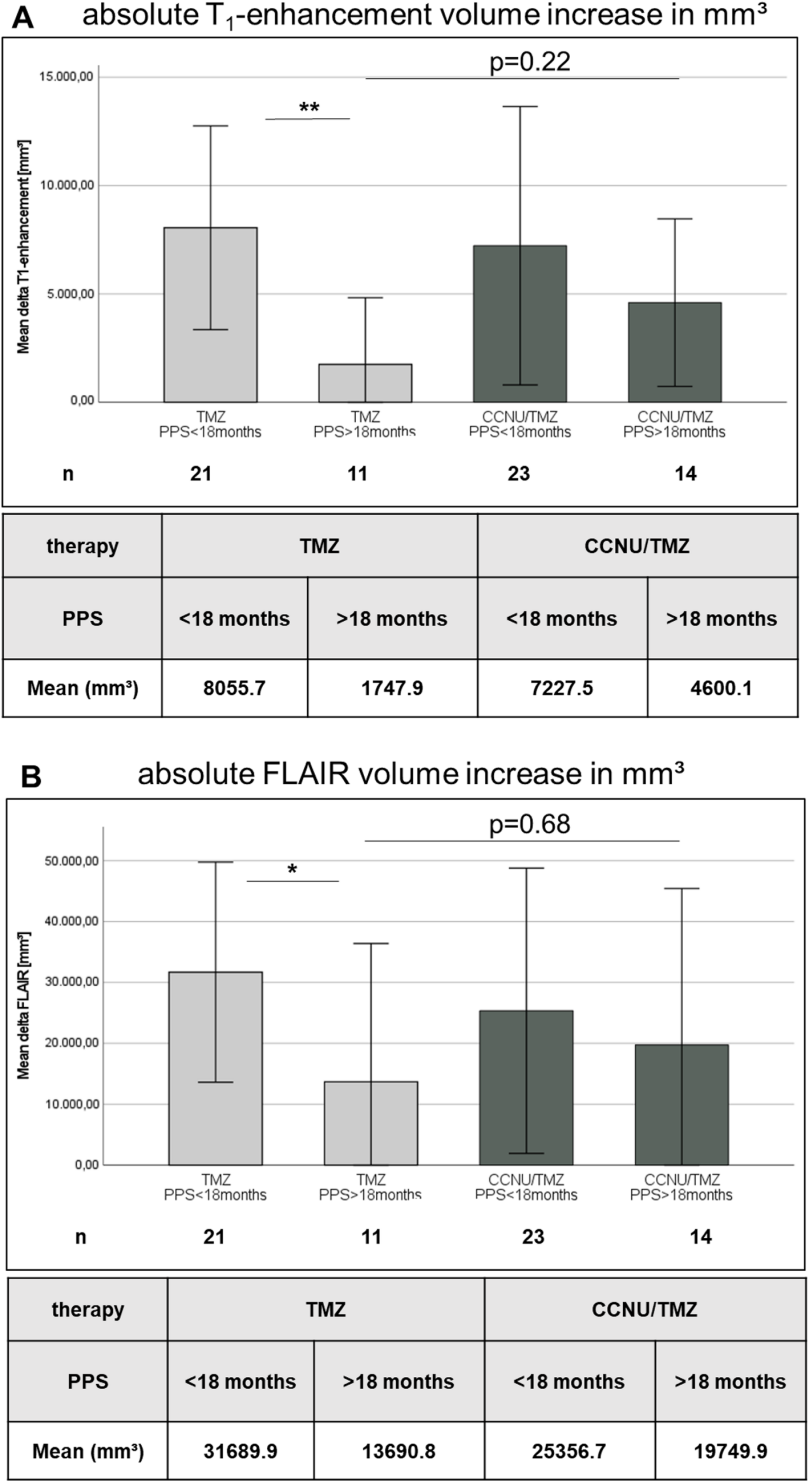
T_1_-enhancement and FLAIR volume increase at mRANO defined progression. For each patient in each group T_1_-enhancement (**A**) and FLAIR (**B**) volume increase from baseline time point to mRANO defined progression time point is given (mm^3^). T_1_-enhancement and FLAIR volume increase patients in TMZ group differed significantly (p = 0.007 and p = 0.03, respectively) but not in CCNU/TMZ group (p = 0.567 and 0.914, respectively). Comparing long PPS groups, CCNU/TMZ tended to a stronger volumetric increase in T_1_ enhancement (p = 0.22). Error bars show 95% confidence interval of Means. Statistical analysis was performed using Mann–Whitney-test. p < 0.05 was considered significant and marked as * =  < 0.05, ** =  < 0.005

Seventy patients (about 80% of the patients that were included in the survival analysis in this study) were evaluable for imaging analysis and constituted the cohort on which all following MRI analyses are based on. Patients with long PPS in the CCNU/TMZ arm showed a tendency to a stronger increase in T_1_-enhancement volume (mean delta 4.600,1 mm^3^ vs. 1.747,9 mm^3^, p = 0.219, Fig. 3A) but not in FLAIR volume (mean delta 19.749,9 mm^3^ vs 13.690,8 mm^3^, p = 0.682, Fig. 3B) at progression as compared to long PPS patients of the TMZ arm. Overall, in the TMZ monotherapy arm, patients with short PPS could be well distinguished from patients with long PPS by their strongly increasing volumes of contrast-enhancement (mean delta 8.055,7 mm^3^ vs 1.747,9 mm^3^, p = 0.005) and FLAIR hyperintense lesions (mean delta 31.689,9 mm^3^ vs 13.690,9 mm^3^, p = 0.03). In contrast, patients with CCNU/TMZ combination therapy showed no difference in T_1_-enhancement increase (mean delta 7.227,5 mm^3^ vs 4.600,1 mm^3^, p = 0.567) or FLAIR increase (mean delta 25.356,7 mm^3^ vs. 19.7489,9 mm^3^, p = 0.914) in short-PPS vs. long-PPS patients. In summary, in the TMZ monotherapy arm, the increase of the contrast-enhancing lesion and FLAIR lesion at mRANO-defined progression was inversely related to OS, whereas in the CCNU/TMZ arm, no such relation was observed. Thus, these parameters do not allow to distinguish patients with long PPS from patients with short PPS in the CCNU/TMZ group at mRANO-defined progression time point.

**Fig. 4:**
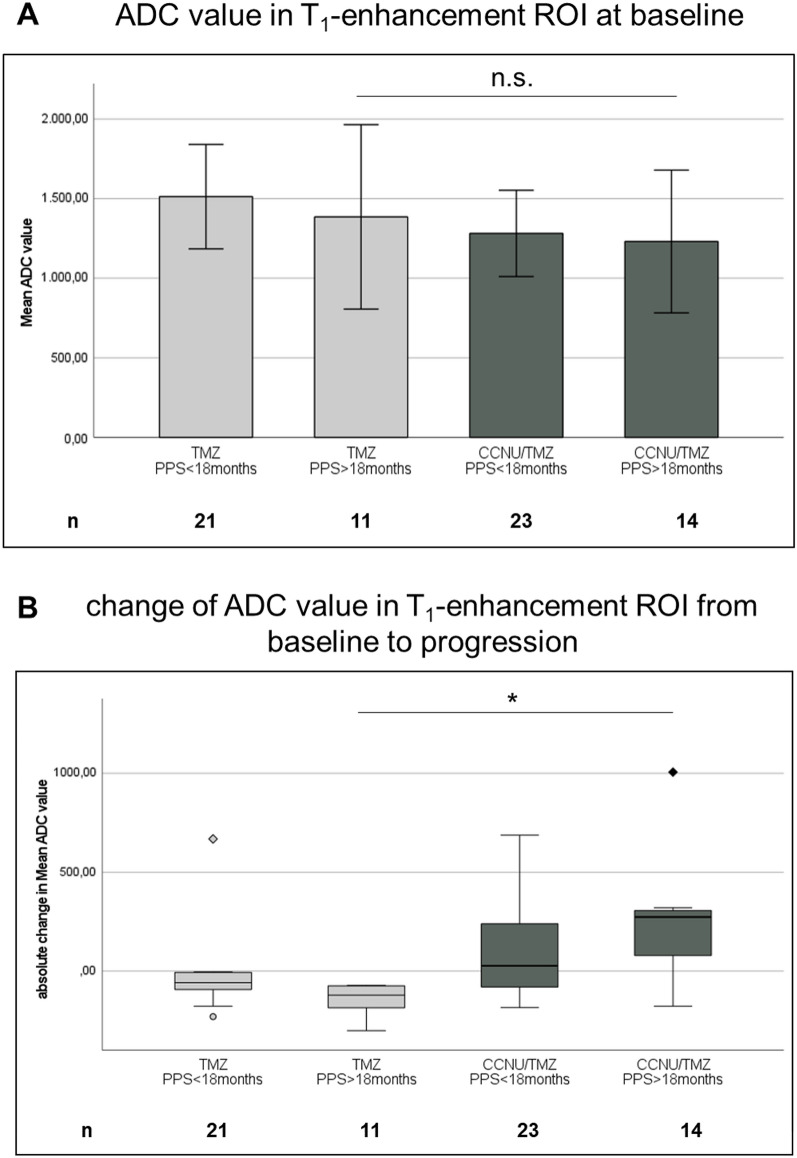
ADC analysis of T_1_-enhancement region of interest (ROI). For each patient in each group Mean ADC value of T_1_-enhancing tumor ROI was calculated. In (**A**) the Mean ADC value of baseline tumor (last prior to progression) is shown. There is no significant difference between long PPS TMZ arm and long PPS CCNU/TMZ arm (p = 0.94, Mann–Whitney test). In (**B**) the absolute change in Mean ADC value of T_1_-enhancement ROI from baseline to progression time point is shown. The change is significantly higher in long PPS CCNU/TMZ arm, comparing to long PPS TMZ arm (p = 0.017, Mann–Whitney test). Error bars show 95% confidence interval of Means. Statistical analysis was performed using Mann–Whitney-test. p < 0.05 was considered significant and marked as * =  < 0.05

Analysis of ADC maps revealed no statistical difference of mean ADC values between treatment groups as a whole at baseline time point (Fig. 4A). However, long PPS patients of the CCNU/TMZ group showed a higher absolute increase in ADC value from baseline (last prior to progression) to mRANO progression time point than long PPS patients of the TMZ group (Fig. 4B, p = 0.017). These data suggest that CCNU/TMZ therapy might lead to changes in tumor structure that can be identified in ADC imaging and may be different from the radiological changes seen in patients after TMZ monotherapy.

## Discussion

In the present study based on data from the prospective CeTeG/NOA-09 trial, we are able to identify a subgroup of CCNU/TMZ-treated patients that have a particularly short first PFS despite long PPS and OS. These patients show MRI features (significant increase in mean ADC values; tendency to a stronger increase in contrast enhancement at time point of mRANO-defined progression) that sets them apart from their counterparts with long PPS in the TMZ monotherapy arm.

This observation is remarkable since it is contradictory to the current literature describing a reliable correlation between PFS and OS in glioblastoma and other malignancies [26, 27]. As the PFS is decreased compared to the long PPS group of TMZ, we assumed that tumor changes in MRI of these patient groups might differ biologically from each other. A possible explanation for this difference may be undetected pseudoprogressions that evade correct diagnosis by mRANO criteria and are falsely diagnosed as progressive disease. As we already know, standard MRI and clinical assessment cannot reliably differentiate pseudoprogression and progressive disease. Even histology can be difficult to interpret, as there are no defined criteria for diagnosing progression, pseudoprogression or mixed forms [28]. So far, we are not able to finally proof the hypothesis of undetected pseudoprogressions but we can rule out some alternative hypotheses such as that an imbalanced distribution of further line therapies or other known prognostic factors (Herrlinger et al., 2019 and, for subgroup comparisons see Table 1 and Supplemantary Fig. 2) that may be responsible for the survival differences.

Our image analyses show that the MRI at time of mRANO-defined progression in the long PPS CCNU/TMZ group is particularly characterized by an increase in ADC values, while ADC value of contrast enhancing tumor is stable in TMZ patients. Current literature describes a higher ADC value as compatible with pseudoprogression rather than progression [16–19]. Thus, our finding might support the hypothesis that pseudoprogressive changes appear to be more frequent and more distinct after CCNU/TMZ therapy. Increase in ADC values could possibly be explained by a variety of biological / histopathological factors, including disintegration of cellular membranes, reduction in cell density and as a result an increase in extracellular space. This pattern is rather observed in pseudoprogression than true progession [29].

The limitations of our analyses are set by the post hoc approach (despite the prospective collection of data in the trial) with MRI data evaluable for volumetric analysis lacking in some of the patients. Further limitations are the small number of patients making detection of small group differences difficult and radiomics approaches impossible, and the lack of histological data. Future imaging analysis in prospective cohorts should also include analysis of MRI perfusion imaging, amino acid positron-emission-tomography (PET) and novel imaging approaches, such as amide proton transfer (APT)-weighted MRI that showed promise results in the differentiation of therapy-related changes and tumor progression [12–15].

Overall, we conclude that the modified RANO criteria might not be entirely suitable for patients with MGMT-methylated glioblastoma receiving CCNU/TMZ treatment. This is in line with reports that pseudoprogression may be substantially prolonged [30, 31] and thus go undetected by strictly applied mRANO criteria. In the context of CCNU/TMZ therapy, we would therefore rather suggest to perform repeat follow-up examinations (ideally complemented by additional imaging techniques such as MRI perfusion imaging or amino acid PET) instead of prematurely stopping an effective treatment or advancing to further lines of therapy.

### Supplementary Information

Below is the link to the electronic supplementary material.

Supplementary file1 (DOCX 698 KB)

## Data Availability

The datasets generated during and/or analysed during the current study are available from the corresponding author on reasonable request.
