# Correction to: Undetected pseudoprogressions in the CeTeG/NOA-09 trial: hints from postprogression survival and MRI analyses

**DOI:** 10.1007/s11060-023-04488-z

**Published:** 2023-11-03

**Authors:** Thomas Zeyen, Daniel Paech, Johannes Weller, Niklas Schäfer, Theophilos Tzaridis, Cathrina Duffy, Louisa Nitsch, Matthias Schneider, Anna-Laura Potthoff, Joachim Peter Steinbach, Peter Hau, Uwe Schlegel, Clemens Seidel, Dietmar Krex, Oliver Grauer, Roland Goldbrunner, Pia Susan Zeiner, Ghazaleh Tabatabai, Norbert Galldiks, Walter Stummer, Elke Hattingen, Martin Glas, Alexander Radbruch, Ulrich Herrlinger, Christina Schaub

**Affiliations:** 1 Division of Clinical Neurooncology, Department of Neurology, University Hospital Bonn, Bonn, Germany; 2 Department of Neuroradiology, University Hospital Bonn, Bonn, Germany; 3 Department of Neurosurgery, University Hospital Bonn, Bonn, Germany; 4 Dr. Senckenberg Institute of Neurooncology, University of Frankfurt, Frankfurt, Germany; 5 Department of Neurology and Wilhelm Sander NeuroOncology Unit, University Hospital Regensburg, Regensburg, Germany; 6 Department of Neurology, Klinik Hirslanden, Zürich, Switzerland; 7 Department of Radiation Oncology, University of Leipzig, Leipzig, Germany; 8 Department of Neurosurgery, Technische Universität Dresden, Faculty of Medicine and University Hospital Carl Gustav Carus, Fetscherstrasse 74, 01307 Dresden, Germany; 9 Department of Neurology, University of Münster, Münster, Germany; 10 Center of Neurosurgery Department of General, Neurosurgery University of Cologne, Cologne, Germany; 11 Department of Neurology and Interdisciplinary Neuro-Oncology, Institute for Clinical Brain Research, University Hospital Tübingen, Eberhard Karls University Tübingen, HertieTübingen, Germany; 12 Center for Neuro-Oncology, Comprehensive Cancer Center Tübingen-Stuttgart, University Hospital Tübingen, Eberhard Karls University Tübingen, Tübingen, Germany; 13 Department of Neurology, Faculty of Medicine and University Hospital Cologne, University of Cologne, Cologne, Germany and Research Center Juelich, Inst. of Neuroscience and Medicine (INM-3), Juelich, Germany; 14 Department of Neurosurgery, University of Münster, Münster, Germany; 15 Department of Neuroradiology, University Hospital Frankfurt, 60590 Frankfurt Am Main, Germany; 16 Division of Clinical Neurooncology, Department of Neurology and Center for Translational Neuro- and Behavioral Sciences (C-TNBS), University Medicine Essen, University Duisburg-Essen, Essen, Germany; 17 German Cancer Consortium (DKTK), Partner Site University Medicine Essen, Hufelandstr. 55, 45147 Essen, Germany

**Correction to: Journal of Neuro-Oncology (2023) 164:607–616** 10.1007/s11060-023-04444-x

In this article Thomas Zeyen and Daniel Paech should have been denoted as equally contributing authors. The original article has been corrected.

