# Supplementary material for: Undetected pseudoprogressions in the CeTeG/NOA-09 trial: hints from postprogression survival and MRI analyses
Source: J Neurooncol. 2023 Sep 20;164(3):607–16. doi: 10.1007/s11060-023-04444-x (PMC10589172; doi:10.1007/s11060-023-04444-x)
Supplement: Supplementary file 1 — Supplementary file1 (DOCX 698 KB) [file 11060_2023_4444_MOESM1_ESM.docx]

**Supplementary figure 1**

**
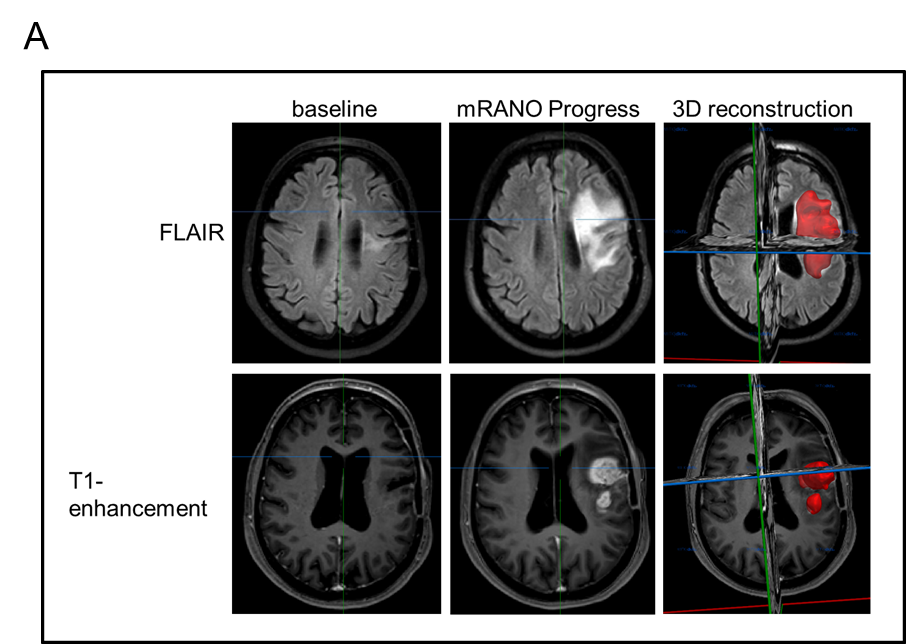
**

**
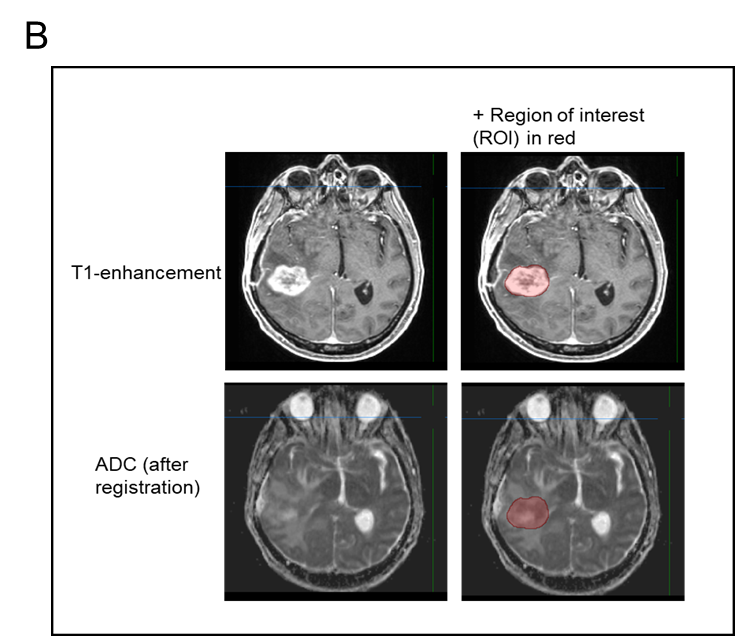
**

**Supplementary figure 1 tumor and edema volumetry** Illustraton shows examplary tumor and edema volumetry and ADC analysis of one patient MRI at baseline and mRANO defined progression time point. 3D reconstruction of progressive tumor is illustrated in red (A). T_1_-enhancement region of interest (ROI) applied to ADC imaging is shown in (B) The tumor was outlined in all axial or coronal planes of MRI data, following interpolation and calculation of tumor volume by MITK calculator (mm³). For more specific information see Methods section.

**Supplementary figure 2**

**
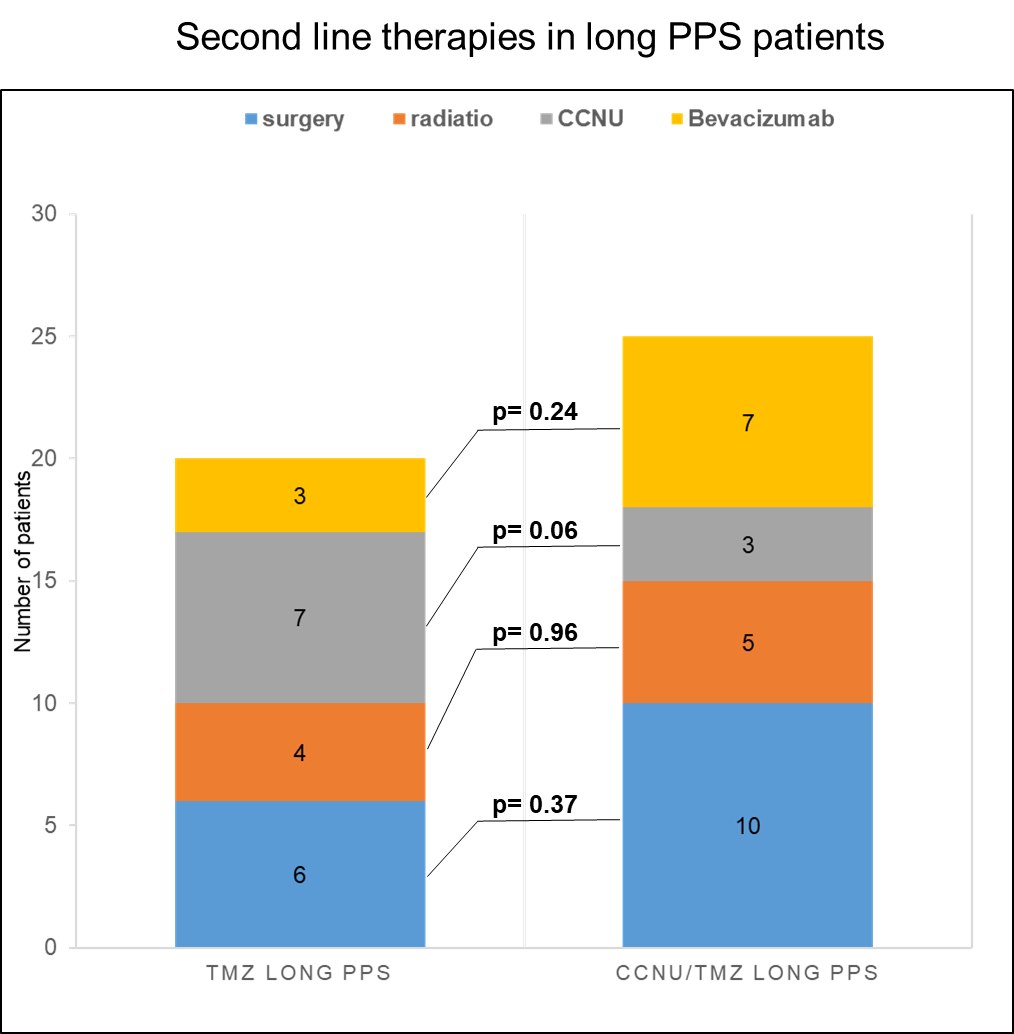
**

**Supplementary figure 2 second line therapies in long PPS patients** Second line therapies in long PPS subgroups are shown. Total numbers of re-surgery, re-radiation, lomustine and Bevacizumab therapy is given. Statistical analysis was performed using chi²-test. P<0.05 was considered significant.
